# Use of simple endoscopic ligation to successfully remove a large torsional colonic lipoma causing intussusception

**DOI:** 10.1055/a-2194-4717

**Published:** 2023-11-03

**Authors:** Yadi Lan, Shulei Zhao, Hongwei Xu

**Affiliations:** 1 Department of Gastroenterology, Shandong Provincial Hospital, Shandong University, Jinan, China; 2 34708 Department of Gastroenterology, Department of Gastroenterology, Shandong Provincial Hospital Affiliated to Shandong First Medical University, Jinan, China

Colonic lipomas are uncommon benign submucosal tumors that are usually asymptomatic. However, some lipomas >4 cm may cause intussusception, abdominal pain, or hematochezia 1 . Most patients with giant lipomas require surgery for relief of symptoms 2 . Recently, endoscopic mucosal resection, endoscopic mucosal resection after precutting, and endoscopic submucosal dissection have been reported for the treatment of large lipomas 3 . However, the fatty tissue is an inefficient conductor of electronic current and may lead to a high incidence of complications 4 . We report the use of endoscopic ligation for the treatment of a large, torsional, colonic lipoma causing intussusception.

A 40-year-old man complained of abdominal pain and hematochezia. Colonoscopy found a huge submucosal mass ( Fig. 1 **a** ), which was considered to be lipoma with adjacent colonic intussusception by computed tomography (CT) ( Fig. 1 **b** ).

**Fig. 1:**
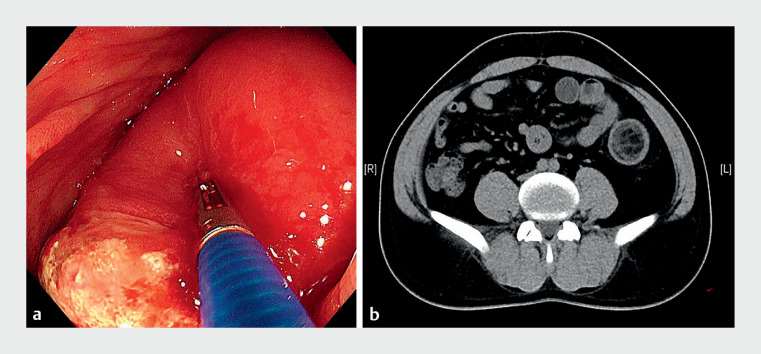
Initial examination. **a** Endoscopic image. **b** Computed tomography image.

Owing to the torsion of the muscularis mucosae and lamina propria layers of the broad lipoma pedicle ( Fig. 2 **a** ), which could not be resolved by submucosal injection, endoscopic submucosal dissection carried a high risk of perforation. Ultimately, we decided to employ endoscopic ligation.

**Fig. 2:**
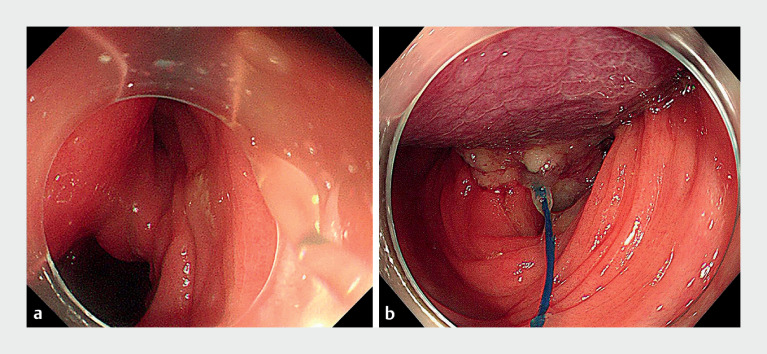
Endoscopic treatment. **a** Torsion of the broad lipoma pedicle. **b** Lipoma after ligation.

First, the nylon loop was gradually tightened and released to ligate the base of the lipoma. The lipoma turned dark purple within 2 minutes ( Fig. 2 **b** ). Six days later, the lipoma detached and embedded in the colon, 20 cm from the anus, and was difficult to remove. On the seventh day, the mass was found in the patient’s stool ( Fig. 3 ) and was confirmed to be a lipoma by histopathology examination, with size 6.5×3.7×3.1 cm. Colonoscopy showed a large ulcer with neat margins and light yellow plaque ( Video 1 ).

**Fig. 3:**
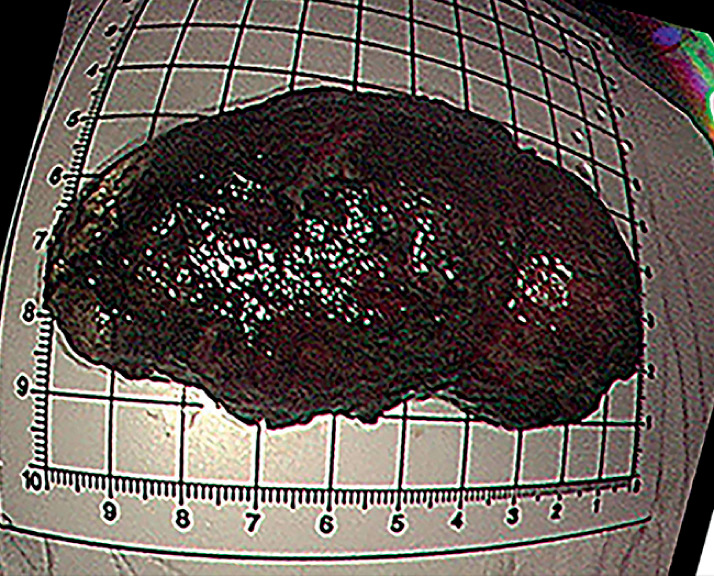
The discharged lipoma.

Use of simple endoscopic ligation to successfully remove a large, torsional, colonic lipoma causing intussusception.Video 1

This case demonstrates the efficacy and safety of endoscopic ligation for giant, torsional, colonic lipoma with broad pedicle. We also thoroughly tracked the patient’s postoperative symptoms ( Fig. 4 ), which provides a reference for similar patients in the future.

**Fig. 4:**
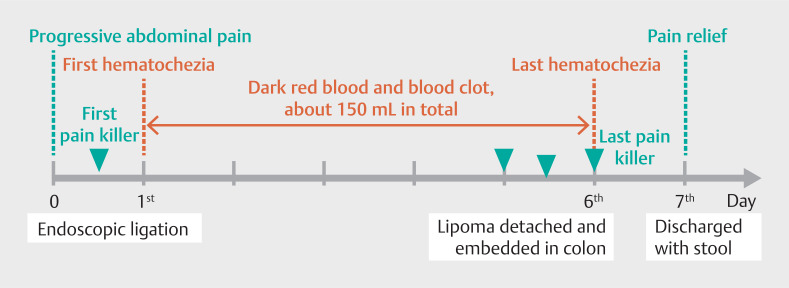
The patient’s postoperative symptoms.

Endoscopy_UCTN_Code_TTT_1AQ_2AD

## Notes

Endoscopy E-Videos https://eref.thieme.de/e-videos
*E-Videos* is an open access online section of the journal *Endoscopy* , reporting on interesting cases and new techniques in gastroenterological endoscopy. All papers include a high-quality video and are published with a Creative Commons CC-BY license. Endoscopy E-Videos qualify for HINARI discounts and waivers and eligibility is automatically checked during the submission process. We grant 100% waivers to articles whose corresponding authors are based in Group A countries and 50% waivers to those who are based in Group B countries as classified by Research4Life (see: https://www.research4life.org/access/eligibility/ ). This section has its own submission website at https://mc.manuscriptcentral.com/e-videos .
